# Supplementary material for: Structures of H5N1 influenza polymerase with ANP32B reveal mechanisms of genome replication and host adaptation
Source: Nat Commun. 2024 May 15;15:4123. doi: 10.1038/s41467-024-48470-3 (PMC11096171; doi:10.1038/s41467-024-48470-3)
Supplement: Supplementary file 1 — Supplementary Information [file 41467_2024_48470_MOESM1_ESM.pdf]

Structures of H5N1 influenza polymerase with ANP32B reveal mechanisms of genome replication and host adaptation

Ecco Staller[1], Loïc Carrique[2], Olivia C. Swann[3], Haitian Fan[1,4], Jeremy R. Keown[2,5], Carol M. Sheppard[3], Wendy S. Barclay[3], Jonathan M. Grimes[2] & Ervin Fodor[1]

[1]Sir William Dunn School of Pathology, University of Oxford, Oxford, UK

[2]Division of Structural Biology, University of Oxford, Oxford, UK

[3]Section of Molecular Virology, Imperial College London, London, UK

[4]Current address: School of Basic Medical Sciences, Zhejiang University School of Medicine, Zhejiang University, Hangzhou, China

[5]Current address: School of Life Sciences, University of Warwick, Coventry, UK

SUPPLEMENTARY FIGURES 1-6

SUPPLEMENTARY TABLES 1 & 2

SUPPLEMENTARY REFERENCES

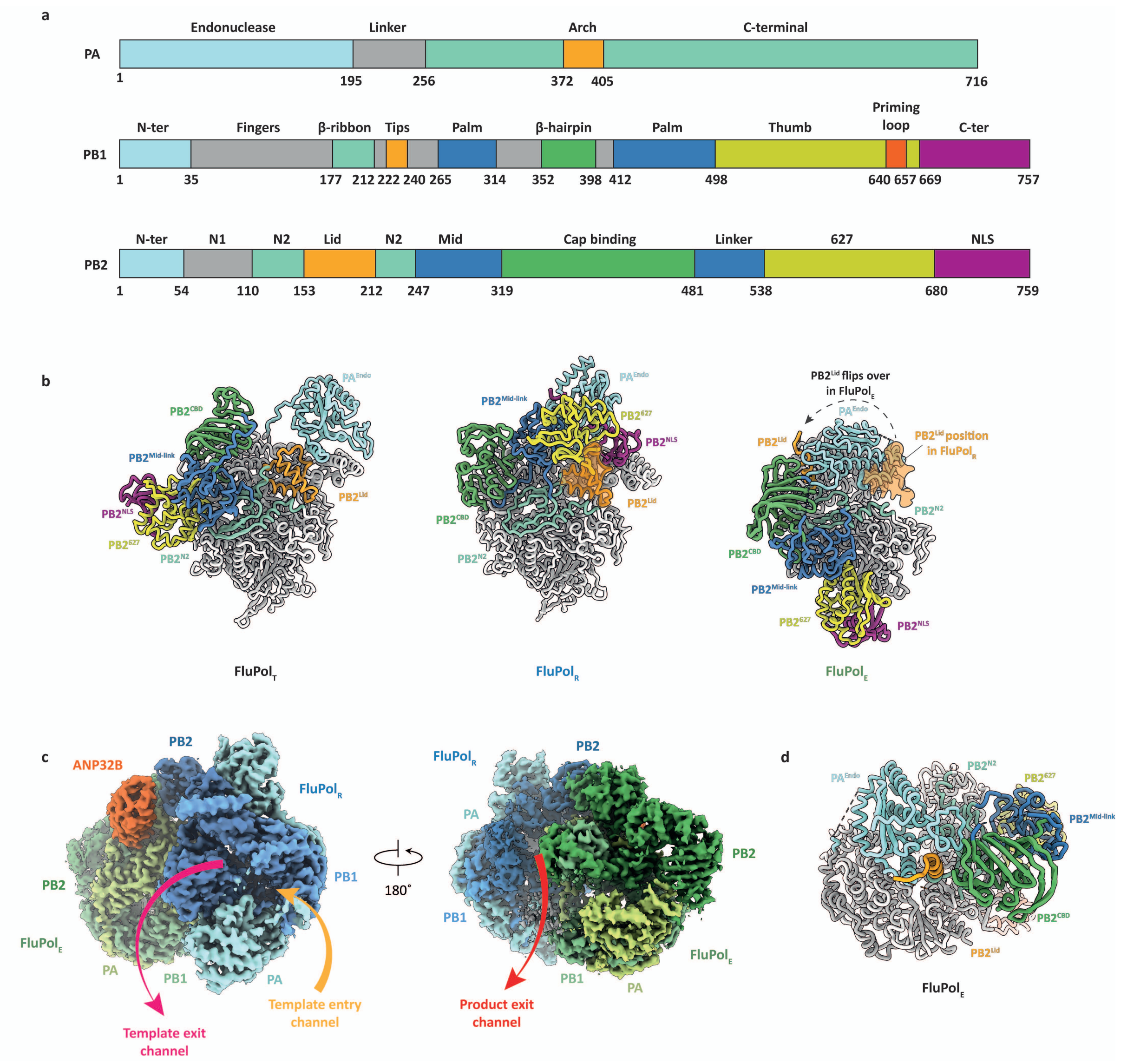

Supplementary Figure 1. Subunits and domain organisation of FluPolA. a, Domain organisation of FluPolA subunits PA, PB1 and PB2. **b,** Structural organization of the flexible PB2 C-terminal domains in the transcriptase (FluPol$_T$, PDB 6RR7), replicase (FluPol$_R$, PDB 8R1J) and encapsidating (FluPol$_E$, PDB 8R1J) conformations. **c**, Cryo-EM map of IAV replication platform highlighting the position of the template entry and exit channels as well as the product exit channel in FluPol$_R$. **d**, Interface between PA^{Endo}, PB2^{Lid} and PB2^{CBD} in FluPol$_E$.

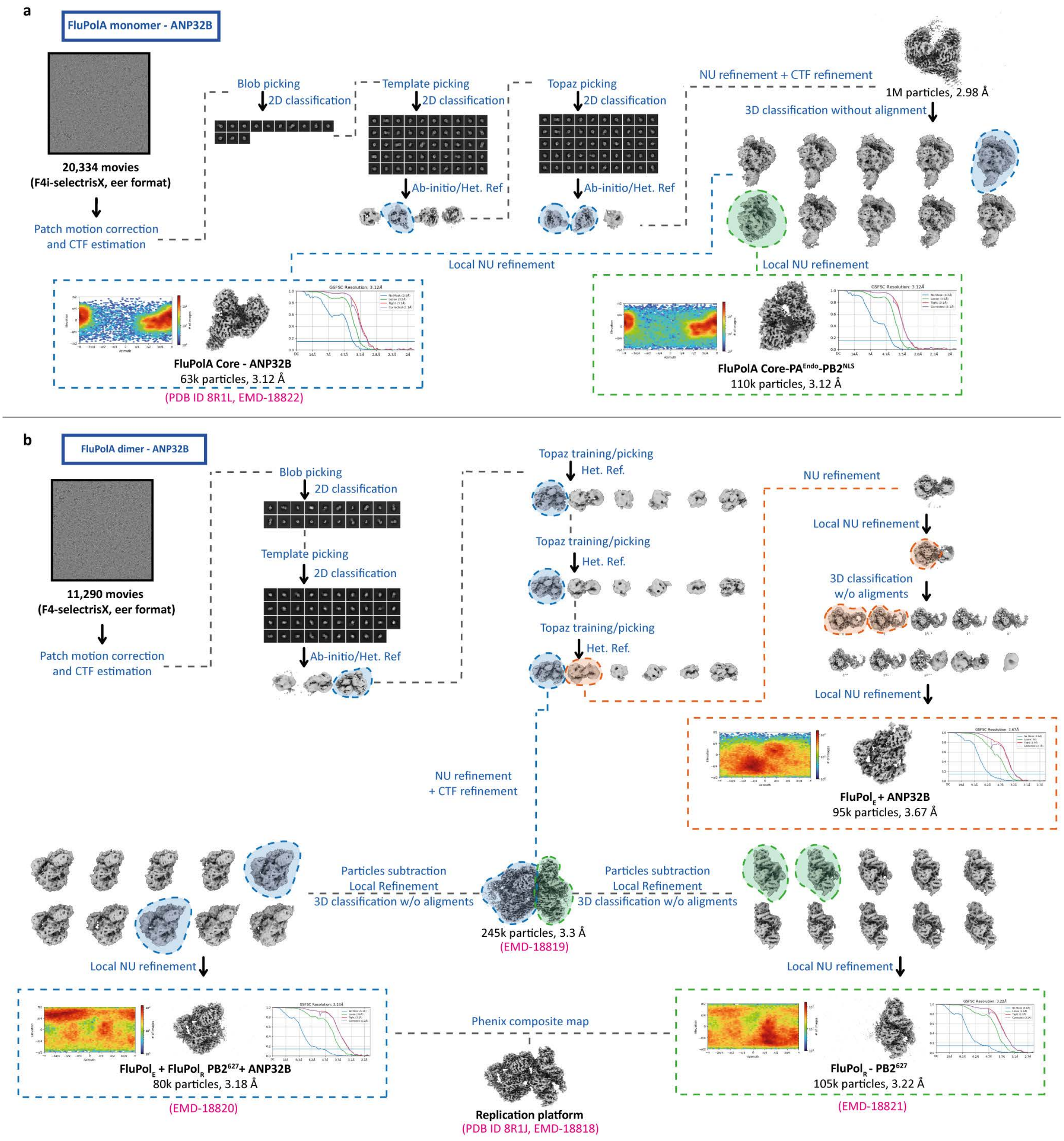

Supplementary Figure 2. Data collection, processing and analysis scheme. a, b, Flowchart for the processing and the classification of the monomeric FluPolA-ANP32B complex (**a**) and dimeric FluPolA-ANP32B complex (**b**).

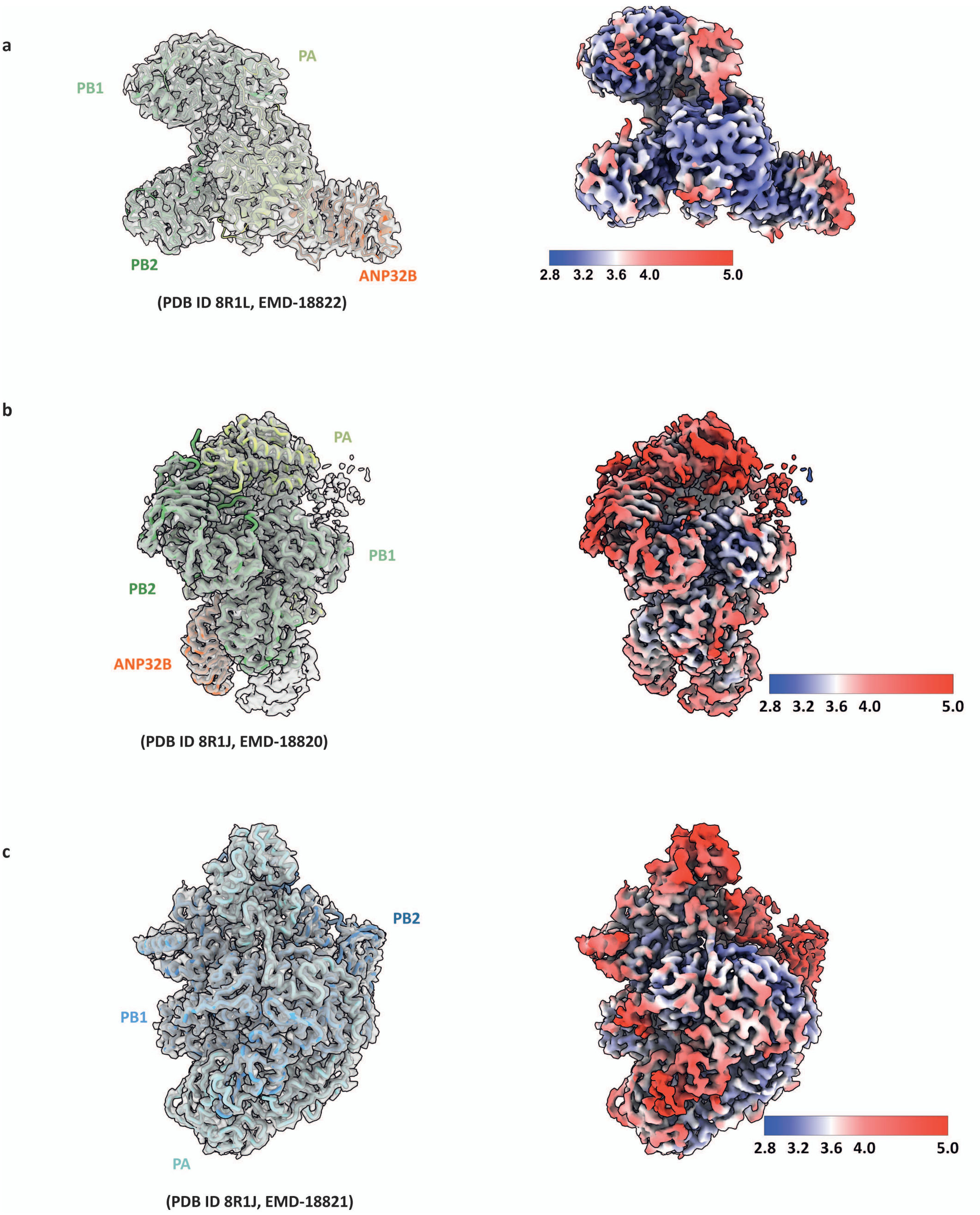

Supplementary Figure 3. Model to map fits and local resolution estimates. a-c, Model to map fits and local resolution estimates for the 3D reconstructions obtained during this study for monomeric FluPolA-ANP32B (**a**) and dimeric FluPolA-ANP32B ($FluPol_E$ + $FluPol_R$-$PB2^{627}$ focused map (**b**) and $FluPol_R$ focused map (**c**)). Local resolutions were computed using CryoSPARC local resolution estimation tool.

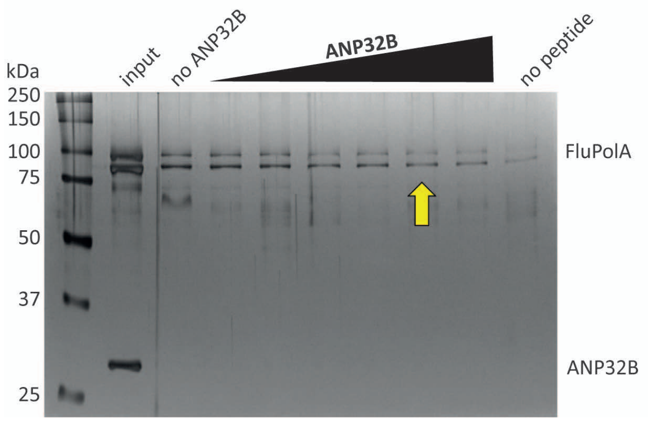

Supplementary Figure 4. Pulldown experiment showing FluPolA (PB1 577E and PA 556R) binding to serine 5 phosphorylated RNAP II CTD peptide in the presence of increasing amounts of ANP32B (range 0.1-fold to 10-fold molar excess relative to FluPolA). The arrow indicates 3-fold molar excess which was selected for subsequent experiments. ANP32B in the input lane depicts 3-fold molar excess. Data represent a single experiment (n=1).

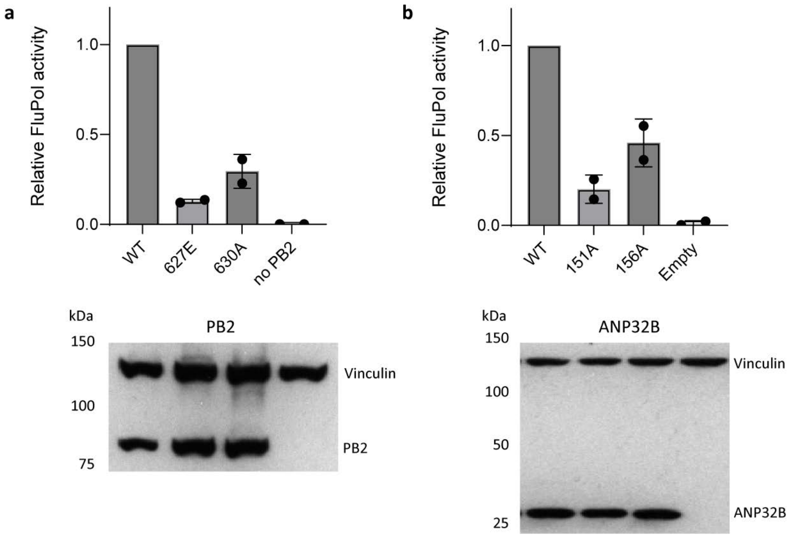

Supplementary Figure 5. Mutations in PB2 and ANP32B affecting the FluPol$_E$-ANP32B interface lead to reduced FluPol activity. a, b, vRNP reconstitution assays were performed in 24-well plates of 293T (**a**) or eHAP TKO (**b**) cells transfected with 0.05 µg pCAGGS plasmids expressing Tky05 PB1 577E, PA 556R, PB2 (as indicated) and NP, with pPolI-firefly luciferase reporter and pcDNA-ANP32B mutants as indicated. Empty pcDNA was co-transfected in negative control wells ('no PB2' and 'Empty') to ensure equal amounts of plasmid DNA in each well. Western blots show expression of PB2 (**a**) and ANP32B (**b**) mutants and Vinculin loading control. Note that the eHAP TKO cells do not express endogenous ANP32B. Data represent two biological repeats (n=2 biologically independent experiments), each in technical triplicate, presented as mean values +/- SD. Statistical significance (confidence interval = 95%) was determined by ordinary one-way ANOVA with Dunnett's multiple comparisons test. Values are compared to the wildtype condition which has been set to 1. P values in (**a**) are: 627E, P=0.0001; 630A, P=0.0003; and no PB2, P<0.0001. P values in (**b**) are: 151A, P=0.0012; 156A, P=0.0052; and Empty, P=0.0005. Source data are provided as a Source Data file.

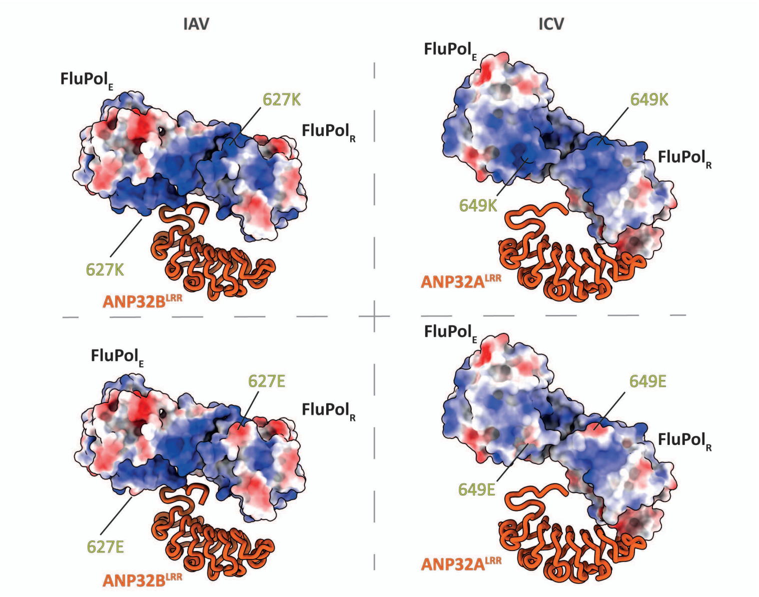

Supplementary Figure 6. Positioning of PB2 residue 627 in FluPol$_E$ and FluPol$_R$ in the context of the IAV replication platform and of the equivalent residue 649 in the ICV replication platform.

	FluPolA monomer - ANP32B<br>EMD-18822<br>8R1L	FluPolA dimer - ANP32B<br>EMD-18818<br>8R1J
**Data collection**		
Microscope	Titan Krios G3i (OPIC)	Titan Krios G3i (OPIC)
Voltage (kV)	300	300
Detector	Falcon 4i - SelectrisX	Falcon 4i - SelectrisX
Recording mode	eer	eer
Magnification	130,000	130,000
Movie/micrograph pixel size (Å)	0.932	0.932
Dose rate (e-/px/sec)	9.37	9.42
Number of frames per movie	60	60
Movie exposure time (s)	4.95	4.9
Total dose (e-/Å^2)	50	50
Defocus range (um)	1.4 to 2.6	1.4 to 2.6
**EM data processing**		
Number of movies/micrographs	20,334	11,290
Box size (px)	300	400
Particle number (total)	2,100,000	1,700,000
Particle number (used in final map)	67,000	105,000
Symmetry	C1	C1
Map resolution (Å, FSC 0.143)	3.12	3.24
Local resolution range (Å, FSC 0.5)	2.78 - 30	2.94 - 30
Map sharpening B-factor (Å^2)	111	85.6
**Model Building and Validation**		
Initial model used	6RR7, AlphaFold model	6RR7, AlphaFold model
Model composition		
Non-hydrogen protein atoms	22132	67795
Protein residues	1383	4238
Nucleotides	/	/
B factors (Å^2) - min/max/mean		
Protein	32.03/123.12/64.49	23.35/156.28/76.74
Nucleotide	/	/
Ligand	/	/
RMSD from ideal		
Bond length (Å)	0.003	0.003
Bond angles (°)	0.505	0.523
Validation		
Molprobity score	1.75	1.89
Clashscore	7.5	7.86
Rotamers outliers (%)	0	0
FSC (0.5) model-vs-map	3.4	3.4
CC model-vs-map (masked)	0.77	0.79
Ramachandran plot		
Favored (%)	95.08	92.77
Allowed (%)	4.92	7.06
Outliers (%)	0	0.17

Supplementary Table 1. Cryo-EM data collection, refinement and validation statistics.

FluPolA subunit	Adaptive mutation	Location	FluPol$_R$-FluPol$_E$ interface	FluPol$_E$-ANP32 interface	IAV subtype	Reference
PB2	T521I	FluPol$_R$			H7N9	Soh *et al* eLife 2019[1]
	K526R	FluPol$_R$			H5N1	Song *et al* Nat Comms 2014[2]
	A588I	FluPol$_R$			H1N1	Lee *et al* Sci Rep 2020[3]
	Q591R	FluPol$_E$			pH1N1	Yamada *et al* Plos Path 2010[4]
	**E627K**	FluPol$_{E/R}$			H2N2	Subbarao *et al* J Virol 1993[5]
	M631L	FluPol$_E$			H9N2	Idoko-Akoh *et al* Nat Comms 2023[6]
	D701N	FluPol$_{E/R}$			H5N1	Gao *et al* Plos Path 2009[7]
	K702R	FluPol$_E$			H5N1	Peacock *et al* J Virol 2023[8]
	S714R	FluPol$_E$			H5N1	Czudai-Matwich *et al* J Virol 2014[9]
PA	E327K	FluPol$_E$			H5N1	Arai *et al* Plos Path 2016[10]
	L336M	FluPol$_E$			H5N1	Welkers *et al* Emerg Microbes Infect 2019[11]
	A343T	FluPol$_E$			H5N1	Arai *et al* Sci Rep 2018[12]
	E349K	FluPol$_E$			H1N1	Chen *et al* Plos Path 2019[13]
	**N383D**	FluPol$_E$			H5N1	Song *et al* Sci Rep 2015[14]
	K385A	FluPol$_E$			H1N1	Liang *et al* Plos One 2012[15]
	D386N	FluPol$_E$			H5N8	Ali *et al* Viruses 2021[16]
	S388R	FluPol$_E$			H5N1	Arai *et al* J Virol 2020[17]
	N409S	FluPol$_E$			H7N9	Yamayoshi *et al* J Virol 2014[18]
	A448E	FluPol$_R$			H5N1	Arai *et al* J Virol 2020[17]
	Q556R	FluPol$_E$			H5N1	Sheppard *et al* Nat Comms 2023[19]
	E613V	FluPol$_R$			H5N1	Arai *et al* Sci Rep 2018[12]
	K615N	FluPol$_R$			H7N7	Gabriel *et al* PNAS 2005[20]

Supplementary Table 2. Overview of mammalian adaptations in avian FluPolA. Adaptations are organised by IAV subunit (PB2 or PA) and whether they are potentially enhancing ANP32 binding or stabilising the replication dimer interface. Adaptations in bold are present in wildtype Tky05 FluPol.

References

1. Soh YS, Moncla LH, Eguia R, Bedford T, Bloom JD. Comprehensive mapping of adaptation of the avian influenza polymerase protein PB2 to humans. *Elife* **8**, (2019).

2. Song W*, et al.* The K526R substitution in viral protein PB2 enhances the effects of E627K on influenza virus replication. *Nature communications* **5**, 5509 (2014).

3. Lee C-Y, An S-H, Choi J-G, Lee Y-J, Kim J-H, Kwon H-J. Rank orders of mammalian pathogenicity-related PB2 mutations of avian influenza A viruses. *Scientific Reports* **10**, 5359 (2020).

4. Yamada S*, et al.* Biological and structural characterization of a host-adapting amino acid in influenza virus. *PLoS pathogens* **6**, e1001034 (2010).

5. Subbarao EK, London W, Murphy BR. A single amino acid in the PB2 gene of influenza A virus is a determinant of host range. *Journal of virology* **67**, 1761-1764 (1993).

6. Idoko-Akoh A*, et al.* Creating resistance to avian influenza infection through genome editing of the ANP32 gene family. *Nature communications* **14**, 6136 (2023).

7. Gao Y*, et al.* Identification of amino acids in HA and PB2 critical for the transmission of H5N1 avian influenza viruses in a mammalian host. *PLoS pathogens* **5**, e1000709 (2009).

8. Peacock TP*, et al.* Mammalian ANP32A and ANP32B Proteins Drive Differential Polymerase Adaptations in Avian Influenza Virus. *Journal of virology* **97**, e0021323 (2023).

9. Czudai-Matwich V, Otte A, Matrosovich M, Gabriel G, Klenk H-D. PB2 Mutations D701N and S714R Promote Adaptation of an Influenza H5N1 Virus to a Mammalian Host. *Journal of virology* **88**, 8735-8742 (2014).

10. Arai Y*, et al.* Novel Polymerase Gene Mutations for Human Adaptation in Clinical Isolates of Avian H5N1 Influenza Viruses. *PLoS pathogens* **12**, e1005583 (2016).

11. Welkers MRA*, et al.* Genetic diversity and host adaptation of avian H5N1 influenza viruses during human infection. *Emerg Microbes Infect* **8**, 262-271 (2019).

12. Arai Y*, et al.* Multiple polymerase gene mutations for human adaptation occurring in Asian H5N1 influenza virus clinical isolates. *Scientific Reports* **8**, 13066 (2018).

13. Chen KY, Santos Afonso ED, Enouf V, Isel C, Naffakh N. Influenza virus polymerase subunits co-evolve to ensure proper levels of dimerization of the heterotrimer. *PLoS pathogens* **15**, e1008034 (2019).

14. Song J, Xu J, Shi J, Li Y, Chen H. Synergistic Effect of S224P and N383D Substitutions in the PA of H5N1 Avian Influenza Virus Contributes to Mammalian Adaptation. *Sci Rep* **5**, 10510 (2015).

15. Liang Y, Danzy S, Dao LD, Parslow TG, Liang Y. Mutational Analyses of the Influenza A Virus Polymerase Subunit PA Reveal Distinct Functions Related and Unrelated to RNA Polymerase Activity. *PloS one* **7**, e29485 (2012).

16. Ali M*, et al.* Genetic Characterization of Highly Pathogenic Avian Influenza A(H5N8) Virus in Pakistani Live Bird Markets Reveals Rapid Diversification of Clade 2.3.4.4b Viruses. *Viruses* **13**, (2021).

17. Arai Y*, et al.* PA Mutations Inherited during Viral Evolution Act Cooperatively To Increase Replication of Contemporary H5N1 Influenza Virus with an Expanded Host Range. *Journal of virology* **95**, (2020).

18. Yamayoshi S*, et al.* Virulence-affecting amino acid changes in the PA protein of H7N9 influenza A viruses. *Journal of virology* **88**, 3127-3134 (2014).

19. Sheppard CM*, et al.* An Influenza A virus can evolve to use human ANP32E through altering polymerase dimerization. *Nature communications* **14**, 6135 (2023).

20. Gabriel G, Dauber B, Wolff T, Planz O, Klenk HD, Stech J. The viral polymerase mediates adaptation of an avian influenza virus to a mammalian host. *Proceedings of the National Academy of Sciences of the United States of America* **102**, 18590-18595 (2005).
