# Supplementary material for: Structures of H5N1 influenza polymerase with ANP32B reveal mechanisms of genome replication and host adaptation
Source: Nat Commun. 2024 May 15;15:4123. doi: 10.1038/s41467-024-48470-3 (PMC11096171; doi:10.1038/s41467-024-48470-3)
Supplement: Supplementary file 3 — Description of Additional Supplementary Files [file 41467_2024_48470_MOESM3_ESM.pdf]

Description of Additional Supplementary Files

File Name: Supplementary Movie 1
Description: An overview of the IAV FluPol-ANP32B replication platform (PDB ID: 8R1J), followed by a comparison with the ICV FluPol-ANP32A complex (PDB ID: 6XZR), highlighting the distinctive arrangement of the flexible domains. The colour scheme is identical to Fig. 2b.
