# Supplementary material for: Structures of H5N1 influenza polymerase with ANP32B reveal mechanisms of genome replication and host adaptation
Source: Nat Commun. 2024 May 15;15:4123. doi: 10.1038/s41467-024-48470-3 (PMC11096171; doi:10.1038/s41467-024-48470-3)
Supplement: Supplementary file 5 — Reporting Summary [file 41467_2024_48470_MOESM5_ESM.pdf]

nature portfolio

Corresponding author(s): Jonathan Grimes, Ervin Fodor

Last updated by author(s): Apr 17, 2024

Reporting Summary

Nature Portfolio wishes to improve the reproducibility of the work that we publish. This form provides structure for consistency and transparency in reporting. For further information on Nature Portfolio policies, see our Editorial Policies and the Editorial Policy Checklist.

Statistics

For all statistical analyses, confirm that the following items are present in the figure legend, table legend, main text, or Methods section.

n/a	Confirmed	
☐	☒	The exact sample size (*n*) for each experimental group/condition, given as a discrete number and unit of measurement
☐	☒	A statement on whether measurements were taken from distinct samples or whether the same sample was measured repeatedly
☐	☒	The statistical test(s) used AND whether they are one- or two-sided<br>*Only common tests should be described solely by name; describe more complex techniques in the Methods section.*
☒	☐	A description of all covariates tested
☐	☒	A description of any assumptions or corrections, such as tests of normality and adjustment for multiple comparisons
☐	☒	A full description of the statistical parameters including central tendency (e.g. means) or other basic estimates (e.g. regression coefficient) AND variation (e.g. standard deviation) or associated estimates of uncertainty (e.g. confidence intervals)
☐	☒	For null hypothesis testing, the test statistic (e.g. *F*, *t*, *r*) with confidence intervals, effect sizes, degrees of freedom and *P* value noted<br>*Give P values as exact values whenever suitable.*
☒	☐	For Bayesian analysis, information on the choice of priors and Markov chain Monte Carlo settings
☒	☐	For hierarchical and complex designs, identification of the appropriate level for tests and full reporting of outcomes
☒	☐	Estimates of effect sizes (e.g. Cohen's *d*, Pearson's *r*), indicating how they were calculated

Our web collection on statistics for biologists contains articles on many of the points above.

Software and code

Policy information about availability of computer code

Data collection	EPU v3.4
Data analysis	GraphPad Prism 10, Image J, cryosparc V4.2, ChimeraX 1.6.1, WinCoot 0.9.8.7, PHENIX 1.19.2

For manuscripts utilizing custom algorithms or software that are central to the research but not yet described in published literature, software must be made available to editors and reviewers. We strongly encourage code deposition in a community repository (e.g. GitHub). See the Nature Portfolio guidelines for submitting code & software for further information.

Data

Policy information about availability of data

All manuscripts must include a data availability statement. This statement should provide the following information, where applicable:

- Accession codes, unique identifiers, or web links for publicly available datasets
- A description of any restrictions on data availability
- For clinical datasets or third party data, please ensure that the statement adheres to our policy

All data are included in the paper, supplementary information or source data; source data are provided with this paper. Structural data generated in this study have been deposited in PDB and EMDB under accession codes PDB 8R1L, EMD-18822 (monomeric FluPolA - ANP32B), PDB 8R1J, EMD-18818 (dimeric FluPolA - ANP32B),

EMD-18819 (consensus map), EMD-18820 (FluPolE + FluPolR-PB2627 focused map) and EMD-18821 (FluPolR focused map). Structural data used in this study are available in the PDB database under accession codes 6FHH, 6XZR and 6RR7.

Research involving human participants, their data, or biological material

Policy information about studies with human participants or human data. See also policy information about sex, gender (identity/presentation), and sexual orientation and race, ethnicity and racism.

Reporting on sex and gender	N/A
Reporting on race, ethnicity, or other socially relevant groupings	N/A
Population characteristics	N/A
Recruitment	N/A
Ethics oversight	N/A

Note that full information on the approval of the study protocol must also be provided in the manuscript.

Field-specific reporting

Please select the one below that is the best fit for your research. If you are not sure, read the appropriate sections before making your selection.

☒ Life sciences ☐ Behavioural & social sciences ☐ Ecological, evolutionary & environmental sciences

For a reference copy of the document with all sections, see nature.com/documents/nr-reporting-summary-flat.pdf

Life sciences study design

All studies must disclose on these points even when the disclosure is negative.

Sample size	Sample sizes were estimated on the basis of previous studies using similar methods and analyses that are widely published, for example see PMID: 26738596, PMID: 31485076, PMID: 33208942, PMID: 35017564.
Data exclusions	Following the first steps of cryo-EM data processing, a small number of the acquired cryo-EM movies were discarded owing to poor ice quality, excessive movement or defocus. Otherwise no data were excluded.
Replication	All functional assays were carried out at least two to three times. All attempts to replicate data were successful.
Randomization	We were not carrying out randomized trials. This is a structure-function analysis. Cryo-EM images were collected automatically in an unbiased manner.
Blinding	This is a structure-function analysis using cryo-EM and functional analysis involving biochemical and cell culture-based studies. Cryo-EM images were acquired automatically. Consequently, blinding is not relevant to this study.

Reporting for specific materials, systems and methods

We require information from authors about some types of materials, experimental systems and methods used in many studies. Here, indicate whether each material, system or method listed is relevant to your study. If you are not sure if a list item applies to your research, read the appropriate section before selecting a response.

Materials & experimental systems

n/a	Involved in the study	
☐	☒	Antibodies
☐	☒	Eukaryotic cell lines
☒	☐	Palaeontology and archaeology
☒	☐	Animals and other organisms
☒	☐	Clinical data
☒	☐	Dual use research of concern
☒	☐	Plants

Methods

n/a	Involved in the study	
☒	☐	ChIP-seq
☒	☐	Flow cytometry
☒	☐	MRI-based neuroimaging

Antibodies

Antibodies used	rabbit anti-ANP32B (Abcam, ab200836); rabbit anti-influenza A virus PB2 (Genetex, GTX125925); rabbit anti-Vinculin (Abcam, ab129002); goat anti-rabbit antibody conjugated to horseradish peroxidase (Genetex, GTX213110-01)
Validation	Validations as described by the manufacturer's:<br>https://www.abcam.com/products/primary-antibodies/phapi2--april-antibody-epr14588-ab200836<br>https://www.genetex.com/Product/Detail/Influenza-A-virus-PB2-protein-antibody/GTX125925<br>https://www.abcam.com/products/primary-antibodies/vinculin-antibody-epr8185-ab129002<br>https://www.genetex.com/Product/Detail/Goat-Anti-Rabbit-IgG-antibody-HRP/GTX213110-01

Eukaryotic cell lines

Policy information about cell lines and Sex and Gender in Research

Cell line source(s)	Sf9 insect cells and HEK 293T cells were sourced from the Cell Bank of the Sir William Dunn School of Pathology, University of Oxford. eHAP cells were purchased from Horizon Discovery and genetically engineered to knock out ANP32A, ANP32B and ANP32E using CRISPR/Cas9 technology, as described in Staller et al J Virol 2019 (https://pubmed.ncbi.nlm.nih.gov/31217244/) and Sheppard et al Nat Commun 2023 (https://pubmed.ncbi.nlm.nih.gov/37816726/).
Authentication	Authentication was not performed for this study.
Mycoplasma contamination	Mycoplasma testing revealed no contamination.
Commonly misidentified lines (See ICLAC register)	No commonly misidentified cell lines were used.

Plants

Seed stocks	N/A
Novel plant genotypes	N/A
Authentication	N/A
